# The Biological Enhancement of Spinal Fusion for Spinal Degenerative Disease

**DOI:** 10.3390/ijms19082430

**Published:** 2018-08-17

**Authors:** Takahiro Makino, Hiroyuki Tsukazaki, Yuichiro Ukon, Daisuke Tateiwa, Hideki Yoshikawa, Takashi Kaito

**Affiliations:** Department of Orthopedic Surgery, Osaka University Graduate School of Medicine, 2-2 Yamadaoka, Suita, Osaka 565-0871, Japan; t-makino@za2.so-net.ne.jp (T.M.); tsukazaki.hiroyuki@gmail.com (H.T.); gonza721ukon@yahoo.co.jp (Y.U.); tateiwa.daisuke1@gmail.com (D.T.); yhideki@ort.med.osaka-u.ac.jp (H.Y.)

**Keywords:** spinal fusion, biological, osteoblast, osteoclast, bisphosphonate, parathyroid hormone, bone morphogenetic protein, receptor activator of nuclear factor κB, stem cell, drug delivery system

## Abstract

In this era of aging societies, the number of elderly individuals who undergo spinal arthrodesis for various degenerative diseases is increasing. Poor bone quality and osteogenic ability in older patients, due to osteoporosis, often interfere with achieving bone fusion after spinal arthrodesis. Enhancement of bone fusion requires shifting bone homeostasis toward increased bone formation and reduced resorption. Several biological enhancement strategies of bone formation have been conducted in animal models of spinal arthrodesis and human clinical trials. Pharmacological agents for osteoporosis have also been shown to be effective in enhancing bone fusion. Cytokines, which activate bone formation, such as bone morphogenetic proteins, have already been clinically used to enhance bone fusion for spinal arthrodesis. Recently, stem cells have attracted considerable attention as a cell source of osteoblasts, promising effects in enhancing bone fusion. Drug delivery systems will also need to be further developed to assure the safe delivery of bone-enhancing agents to the site of spinal arthrodesis. Our aim in this review is to appraise the current state of knowledge and evidence regarding bone enhancement strategies for spinal fusion for degenerative spinal disorders, and to identify future directions for biological bone enhancement strategies, including pharmacological, cell and gene therapy approaches.

## 1. Introduction

Spinal arthrodesis is one of the most common surgical procedures used for the treatment of various spinal pathologies, such as spinal deformity, spondylolisthesis, foraminal stenosis, or disc disease. A nationwide epidemiological study in the United States reported a 2.4-fold increase in the number of spinal fusion surgeries performed between 1998 and 2008 [1]. Various techniques were reported for performing spinal arthrodesis, including different surgical approaches, graft materials used, and the instrumentation method. However, whichever spinal arthrodesis technique is performed, the fundamental aim is to achieve bony fusion at a mobile segment after the transplantation of autologous, allogeneic or artificial bone graft, and to induce bone modeling and remodeling. The insufficiency of bony fusion or pseudoarthrosis/non-union after spinal arthrodesis can cause a loss of correction and instrumentation failure or deterioration of patients’ quality of life (QOL) [2,3,4,5,6]. Thus, early and successful bony fusion can provide better radiological and clinical outcomes. 

In many countries, the segment of the general population over the age of 60 years have been continuously growing [7]. In these aging societies, the prevalence of degenerative spinal disorders is increasing, and the number of older patients who undergo spinal fusion surgeries is also increasing. Deyo et al. [8] reported that the rates of lumbar fusion surgery among patients over the age of 60 years have increased by 230% between 1988 and 2001 in the United States. Rajaee et al. [1] also reported that the rate of spinal fusion surgery among patients over the age of 65 years has increased by 239.2% between 1998 and 2008 in the United States. In these older individuals, low bone quality or osteoporosis is a great concern for achieving bone fusion after spinal arthrodesis. Instrumentation failure and the low osteogenic quality of autologous bone grafts due to osteoporosis may prevent achieving bone fusion.

**Figure 1:**
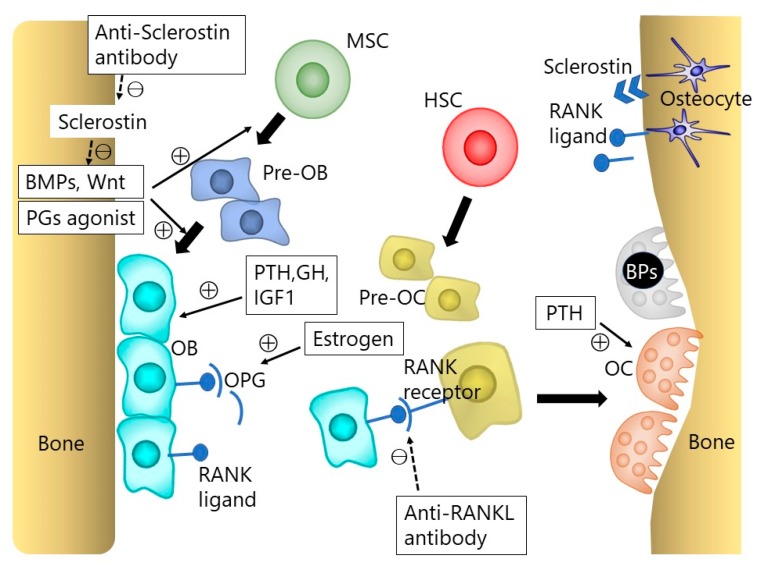
The osteoblast (OB) and osteoclast (OC) lineage cells. Bone homeostasis is maintained by the interaction between osteoblasts, osteoclasts, and osteocytes. Osteoblasts arise from mesenchymal stem cells (MSCs), and osteoclasts from hematopoietic stem cells (HSCs). Osteoblasts can also become osteocytes. Bone morphogenetic proteins (BMPs) and Wnt signaling play an important role in osteoblastogenesis. The receptor activator of nuclear factor κB ligand (RANKL)-RANK interaction is essential for osteoclast differentiation. The RANKL produced by osteoblasts and osteocytes binds to RANK on the osteoclast precursor cells, which triggers the differentiations into osteoclasts. Osteoblast lineage cells also express osteoprotegerin (OPG), which is a soluble decoy receptor of RANKL, blocking RANKL by binding to its cellular receptor RANK. This RANKL-RANK-OPG system plays an important role in bone homeostasis. BP indicates bisphosphonate; GH, growth hormone; IGF1, insulin-like growth factor 1; PG, prostaglandin; PTH, parathyroid hormone.

The process of bone fusion after spinal arthrodesis relies principally on bone remodeling, following adequate bone grafting at fusion sites to prove the scaffold. This process progresses through a complex bone metabolism and relies heavily on osteoblasts, osteoclasts, and osteocytes, with the balance of activity between these two cell types being auto-regulated by metabolic, endocrine, and mechanical signaling pathways, similar to the fracture healing process (Figure 1) [9,10,11]. Boden et al. [12] reported that the histological bone fusion process of posterolateral fusion in rabbits, stating that the membranous bone formation began primarily, and increased volume of woven bone and endochondral ossification were seen subsequently at the bone grafted site. As for vertebral interbody fusion, the local environment of intervertebral space is originally hypo-vascular and of a low nutrient condition. Furthermore, this unfavorable environment becomes exacerbated by degenerative changes. Therefore, the accomplishment of bone fusion at intervertebral space is more demanding biologically compared to posterolateral fusion.

**Figure 2:**
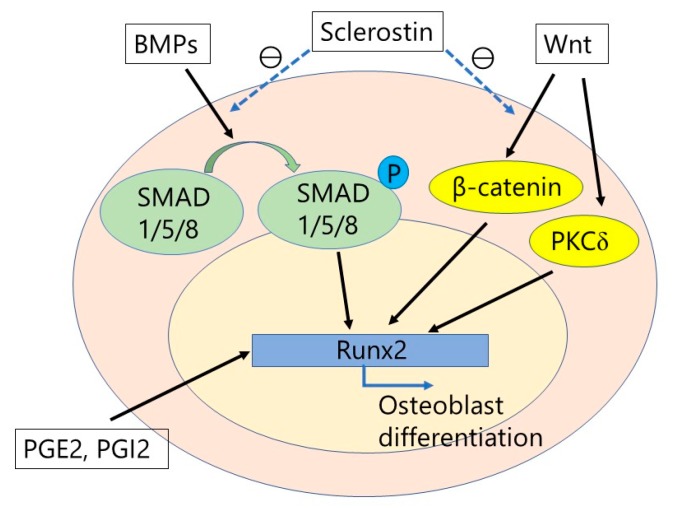
The integration of bone morphogenetic proteins (BMPs) and Wnt signaling. BMPs stimulate osteoblast differentiation by activation of Runx2 via SMAD proteins. Wnt signaling also stimulates osteoblast differentiation by activation of Runx2 through either β-catenin stabilization or protein kinase Cδ (PKCδ). Prostaglandins (PGs), particularly PGE2 and PGI2, also activates Runx2, which results in osteoblast differentiation. In contrast, sclerostin inhibits BMP signaling and Wnt/β-catenin signaling. Therefore, the anti-sclerostin antibody can stimulate osteoblast differentiation.

Osteoblasts are derived from undifferentiated mesenchymal cells. Runt-related transcription factor 2 (Runx2), also described as the core-binding factor subunit alpha-1 (Cbfa1), is necessary to differentiate osteoblasts from undifferentiated mesenchymal precursor cells [13]. Bone morphogenetic proteins (BMPs), Wnt, and the Notch signaling pathways all play important roles in the differentiation of osteoblasts, by regulating the transcription of Runx2 [14,15,16]. BMP signaling, particularly by BMP2 and BMP4, stimulates osteoblast differentiation and function by the activation of Runx2 via SMAD1/5/8. Wnt signaling also stimulates osteoblast differentiation by the activation of Runx2 through either β-catenin stabilization or protein kinase Cδ (PKCδ) (Figure 2). In contrast, Notch signaling inhibits the activity of Runx2 and osteoblast differentiation. Besides these local regulatory signal pathways, osteoblast lineage cell development is also regulated by systemic signals such as Leptin, the parathyroid hormone (PTH), growth hormone, or insulin-like growth factor 1, and sex hormones.

Osteoclasts are giant multinucleated cells which resorb the calcified matrix by secreting acids and collagenolytic enzymes. Osteoclasts are differentiated from hematopoietic cells. The bone marrow is considered to be the site of osteoclast generation, whereas the exact process of osteoclast generation in vivo is still unclear [17]. The receptor activator of nuclear factor κB ligand (RANKL)-RANK interaction is essential for osteoclast differentiation. The RANKL produced by osteoblasts and osteocytes binds to RANK on the osteoclast precursor cells, which triggers the differentiations into osteoclasts. Osteoblast lineage cells also express osteoprotegerin (OPG), which is a soluble decoy receptor of RANKL by blocking RANKL binding to its cellular receptor RANK [18]. Thus, the overexpression of OPG inhibits osteoclastogenesis by RANKL-RANK interaction. This RANKL-RANK-OPG system plays an important role in bone homeostasis through osteoclast regulation (Figure 1) [19].

Bone fusion after spinal arthrodesis can be achieved when the balance of bone homeostasis shifts into an increased bone formation and reduced resorption at the site of bone grafting, though many factors such as age, sex, the spinal fusion procedure, and pre-existing co-morbidities can affect the progress of bone fusion clinically. Therefore, promoting osteoblast activity and/or inhibiting osteoclast activity through the use of biological agents is a feasible approach for promoting successful fusion after spinal arthrodesis, particularly for osteoporotic patients, in addition to the development of biomaterials with high osteogenic properties and improvement in spinal operative and instrumentation techniques. This review is designed to appraise the current methods, and future directions, for the biological enhancement of spinal fusion for degenerative spinal disorders, including pharmacological, cell and gene therapy approaches (Table 1). 

## 2. Bisphosphonates

### 2.1. Mechanism of Action

Bisphosphonates are pyrophosphate analogs that strongly bind to hydroxyapatite and have been shown to reduce the bone turnover rate, increase the bone mineral density (BMD), and prevent fragility fractures [20,21,22]. Bisphosphonates are classified into 2 groups, non-nitrogen-containing and nitrogen-containing, which work differently in bone metabolisms. Etidronate, clodronate, and tiludronate are non-nitrogen-containing bisphosphonates which inhibit adenosine triphosphate (ATP) in cellular metabolism and, therefore, lead to the apoptosis of osteoclasts (Figure 1) [23]. They not only decrease bone resorption but also calcification and, therefore, their long-term use is a potential risk for osteomalacia [24]. On the other hand, pamidronate, alendronate, risedronate, ibandronate, and zoledronate are nitrogen-containing bisphosphonates which have 1000 times more antiresorptive potencies than non-nitrogen-containing bisphosphonates [25]. Nitrogen-containing bisphosphonates block farnesyl pyrophosphate synthase, which is an enzyme of the mevalonate pathway that inhibits protein prenylation and results in the inhibition of the ruffled border formation [26,27]. It has been long discussed whether bisphosphonates help or harm the bone healing process [28], and the effect of bisphosphonates for spinal fusion has also been controversial.

### 2.2. Experimental Studies in Animal Models of Spinal Fusion

There have been many studies regarding the efficacy of bisphosphonate on spinal fusion in animal models. Several authors have reported on the negative effect of alendronate on the progression of spinal fusion [29,30]. Nakao et al. [31] subcutaneously administered alendronate to ovariectomized rats and showed that alendronate inhibited osteoclasts activity around the bone graft. On histology, the ingrowth of newly developed bone was also found to be greater in rats with alendronate than those without. Other studies have provided evidence of a positive effect of bisphosphonates for spinal arthrodesis. Yasen et al. [32] performed spinal fusion in ovariectomized rats and administered zoledronate at various concentrations after surgery. They found that zoledronate did not accelerate the spinal fusion at the clinical dose or lower, but did increase the fusion rate significantly at doses higher than the clinical dose. 

### 2.3. Clinical Trials for Human Spinal Fusion

In a clinical trial of patients with osteoporosis, alendronate reportedly increased the fusion rate and decreased the risk of cage subsidence and postoperative vertebral compression fractures after spinal fusion surgery [33]. A recent study demonstrated the clinical usefulness of zoledronate, which has a 10-fold higher potency in preventing bone loss than alendronate in the ovariectomized rat model [34]. Furthermore, several authors have reported that zoledronate shortened the duration of time to fusion and improved the clinical and radiological outcomes [35,36]. Tu et al. [36] reported that solid fusion after spinal arthrodesis was achieved in 75% of patients who received an intravenous injection of zoledronate, compared to a rate of 56% in those who did not receive zoledronate. Therefore, these authors proposed that zoledronate could reduce the incidence of the subsequent vertebral compression fractures, pedicle screw loosening and cage subsidence at the 2-year follow up. While these studies demonstrate a positive effect of bisphosphonates, Buerba et al. [37] concluded that there were no statistically significant differences in the fusion rate and screw loosening between patients treated with bisphosphonates and those without after spinal arthrodesis in their review. 

### 2.4. Side Effects

In 2003, a first case report was published describing bisphosphonate-related osteonecrosis of the jaw (BRONJ) [38]. The pathology of BRONJ is uncertain, but Santini et al. [39] found that bisphosphonates could lead to osteonecrosis through its effects on blood vessels in the bone by inhibiting the vascular endothelial growth factor. Rasmusson et al. [40] reviewed that exposed bone and subsequent bacterial contamination, typically after dental extraction, seem to trigger BRONJ. Atypical femoral fracture is another serious adverse effect of bisphosphonate, with the risk of atypical femoral fracture increasing as a function of the duration of with bisphosphonate therapy [41].

## 3. Anti-RANKL Monoclonal Antibody

### 3.1. Mechanism of Action

RANKL and its co-stimulatory signals, as well as macrophage colony-stimulating factor, can mediate osteoclastogenic signals. For example, osteopetrosis which is characterized by a high bone mass and a defect in bone-marrow formation can be induced by the congenital lack of osteoclasts [42]. Denosumab, a fully human monoclonal antibody to RANKL, interferes with the interaction between RANKL and RANK receptor on osteoclasts and osteoclast precursors by binding RANKL. Thus, denosumab reversibly inhibits osteoclast-mediated bone resorption (Figure 1) [43]. Denosumab has been recently used for the treatment of severe osteoporosis and its effect on the increase in BMD was reportedly larger than that of bisphosphonates [44,45]. Although bisphosphonates and denosumab are both classified as bone-modifying agents that particularly target osteoclast activity, some differences between these two agents. Kostenuik et al. [46] reported that denosumab significantly reduces cortical porosity compared to bisphosphonates. This difference in bone structure between bisphosphonates and denosumab is attributed to the fact that denosumab acts without binding to bone surfaces, unlike bisphosphonates which are absorbed into bone surfaces [47]. 

### 3.2. Side Effects

Significant and serious side effects of denosumab include hypocalcemia, osteonecrosis of the jaw and atypical femoral fracture, these side effects being similar to those of bisphosphonate [48,49,50]. Zhou et al. [51] reported that denosumab significantly reduced the risk for fractures except for vertebral fractures. In contrast, they also reported that denosumab could increase the risk of serious adverse events related to infection in postmenopausal women with osteoporosis compared to a placebo group; however, there was no significant difference with regard to safety between denosumab and bisphosphonates.

### 3.3. Experimental Studies in Animal Models of Spinal Fusion and Clinical Trials for Human Spinal Fusion

There has been no report on the use of denosumab in a clinical trial for enhancing bone fusion in spinal surgery. However, denosumab has the potential to enhance spinal fusion through its dual effect in inhibiting bone resorption and promoting bone formation. Further studies need to clarify whether denosumab could play a positive role in spinal fusion.

## 4. PTH

### 4.1. Mechanism of Action

PTH is an 84-amino acid polypeptide that is secreted by the parathyroid glands in response to a decrease in plasma calcium. The regulation of serum calcium is the major effect of PTH, which acts directly on osteoblasts, as well as indirectly increasing the differentiation and function of osteoclasts through its interaction with the RANKL of an osteoblast with the RANK receptor of an intermediate osteoclast cell. Finally, PTH enhances the release of calcium by bone resorption. Thus, PTH is involved in bone remodeling, which is an ongoing process where mature bone tissue is removed by osteoclasts (bone resorption) and new bone tissue is formed by osteoblasts (bone formation) (Figure 1). Teriparatide is a recombinant deoxyribonucleic acid form of PTH that has an identical sequence to the biologically active region on the skeleton (the first N-terminal 34 amino acids: rhPTH1-34). 

It has been well known that the continuous infusion of PTH1-34 is associated with a catabolic effect, but that an intermittent administration promotes an anabolic effect on bone [52,53,54]. The mechanism underlying the anabolic and catabolic effect of PTH1-34 on bone metabolism is still unclear. However, we do know that the intermittent exposure to PTH1-34 induces expression of interleukin-11 which, in turn, suppresses Dickopf and, consequently, activates the Wnt signal pathway [16]. Therefore, the expression of bone-formation markers increases before that of bone-resorption markers with intermittent PTH1-34 administration [55,56]. Horwitz et al. [57] developed a seven-day continuous infusion model of PTH1-34 in healthy human adult volunteers and demonstrated that the continuous exposure to PTH1-34 in vivo activated bone resorption. On the other hand, numerous studies have reported on the possible benefit of an intermittent program of administration of PTH1-34. In an experimental animal model, Sato et al. [58] demonstrated, using cortical bone analyses after intermittent PTH1-34 administration in aged ovariectomized rats, that PTH1-34 stimulated the endosteal and periosteal bone formation, with a resulting increase in cortical thickness, a moment of inertia, strength, and stiffness of the femur. Jerome et al. [59] further demonstrated that intermittent PTH1-34 administration increased cancellous bone volume and improved trabecular architecture in ovariectomized cynomolgus monkeys. 

Regarding the frequency of administration, the daily but not weekly administration of PTH1-34 caused cortical porosity and endosteal naïve bone formation in a rabbit model [60,61]. In a clinical study, the EUROFORS study, Graff et al. [62] demonstrated that a daily intermittent PTH1-34 administration increased most vertebral microstructural variables and BMD. Furthermore, several clinical studies showed that the treatment of osteoporosis with daily intermittent PTH1-34 administration decreased the risk of fractures and increased BMD [63,64]. As well, several recent reports have revealed an enhancement of bone healing via the anabolic effect of PTH1-34. In animal experimental models, the intermittent PTH1-34 treatment increased callus formation and accelerated bone healing, which resulted in an increase of the mechanical strength of healed bones [65,66]. Zhang et al. [67] demonstrated that weekly PTH1-34 injections promoted bone fracture healing to the same extent as daily injections in a rat model. Furthermore, Andreassen et al. [68] reported an increase in the guided bone regeneration of calvarial bone defects in a rat model with a daily intermittent PTH1-34 administration. A randomized double-blind placebo-controlled study reported the shortening of the time-to-healing of distal radial fractures, after closed reduction and immobilization, using a daily dose of PTH1-34 compared to a placebo group [69]. 

### 4.2. Experimental Studies in Animal Models of Spinal Fusion 

Several reports have been published regarding the effect of intermittent PTH1-34 on spinal fusion in animal models. Abe et al. [70] reported an enhancement of graft bone healing by intermittent administration of PTH1-34 in a rat model of spinal arthrodesis with autograft. The intermittent administration of PTH1-34 was also reported to improve the fusion rate and decrease the time required for bone graft healing, in the same rat model, providing a structurally superior fusion mass [71]. O’Loughlin et al. [71] reported on the effect of a daily administration of PTH1-34 in a rabbit model of posterolateral spinal fusion with autograft and showed that intermittent PTH1-34 administration promoted a successful fusion using volumetric and histological analyses. Lehman et al. [72] also confirmed an increase in the rate of histological fusion of 86.7% with the intermittent PTH1-34 administration in a rabbit model of posterolateral spinal fusion model, compared to the control autograft only control group (50%). Moreover, there was a strong trend of the superior rate of radiological fusion (85.7%) with PTH1-34 compared to the calcitonin group (56.3%). 

### 4.3. Combination Therapy of PTH1-34 and Anti-RANKL Monoclonal Antibody

The combination therapy of denosumab and PTH1-34 has been considered to be effective due to the potential effect of this combination in inhibiting bone resorption and promoting new bone formation, even in spinal arthrodesis. In an ovariectomized mouse model, a significant increase in BMD of the distal femur and femoral shaft was reported with the use of denosumab and PTH1-34 compared to the use of only denosumab [73]. Tsai et al. [74] evaluated the outcomes of a 2-year program of combined administration of denosumab and PTH1-34 in postmenopausal women with osteoporosis, showing that concomitant PTH1-34 and denosumab therapy increased BMD to a greater extent than either medication used individually. Kitaguchi et al. [75] demonstrated the positive effects of combination therapy of PTH1-34 and denosumab on bone defect regeneration in mice, with this combination accelerating the regeneration of cancellous bone in bone defects in the early phase of bone regeneration and increasing the cancellous bone mass more effectively than either agent individually used. Such a combination therapy could offer a positive impact on spinal arthrodesis, even in humans.

### 4.4. Clinical Trials for Human Spinal Fusion

Several clinical studies on the role of PTH1-34 for spinal fusion have been reported. In their prospective study, Ohtori et al. [76] reported a shorter average delay to fusion after lumbar posterolateral fusion in women with postmenopausal osteoporosis with PTH1-34 than with the use of bisphosphonates. In a further study on spinal fusion among postmenopausal women with osteoporosis, the same authors reported a significantly lower incidence rate of pedicle screw loosening in the subgroup treated with the daily administration of PTH1-34, compared to the risedronate or no medication groups [77]. In a retrospective case series analysis, the authors further confirmed that daily PTH1-34 administration for a period of >6 months was effective in promoting bone union after lumbar posterolateral fusion surgery and, therefore, decreasing the period of treatment [78]. Cho et al. [79] compared the effect of PTH1-34 and bisphosphonate administration on posterior lumbar interbody fusion in patients with osteoporosis through a prospective cohort study and concluded that there was no significant improvement in the overall fusion rate at 24 months after surgery and clinical outcome between the two groups, although the PTH1-34 group showed faster bony union than the bisphosphonate group. Ebata et al. [80] reported that bone fusion, evaluated on CT images, after posterior or transforaminal lumbar interbody fusion in patients with osteoporosis was significantly higher in patients using PTH1-34 than the no PTH1-34 group, both at 4 and 6 months postoperatively. Although the positive effect of PTH1-34 on bone fusion has been described in both animal models and clinical studies, the specific effect of PTH1-34 for bone fusion after spinal arthrodesis remains to be fully clarified. 

## 5. BMP

### 5.1. Mechanism of Action

BMPs are a family of dimeric growth factors that belong to the transforming growth factor superfamily and are critical for skeletal development and bone formation. In 1965, Urist [81] was the first to report on the activity of BMPs as proteins present in the demineralized bone matrix that are capable of osteoinduction in ectopic sites in rats. However, it was not until the late 1980s that the first BMPs were characterized and cloned [82]. Since then, several BMP family members have been isolated, and to date, approximately 20 BMPs have been discovered. Of all, BMP2 and BMP7 significantly induce bone and cartilage formation. While BMP4, BMP5, BMP6, BMP8, BMP9, and BMP10 also contribute to bone formation, BMP3 and BMP13 are BMP inhibitors [83]. BMPs initiate their signaling transduction by binding to a heterodimeric complex of two transmembrane serine–threonine kinase receptors, BMP receptor type I (BMPRI) and type II (BMPR II). Activated receptors phosphorylate SMAD1, 5, and 8, which are specific for the BMP signaling pathway. Then heterodimeric complexes are formed by the phosphorylated SMADs with SMAD4 in the nucleus and regulate the transcription of target genes (Figure 1 and Figure 2) [84].

### 5.2. Clinical Trials for Human Spinal Fusion

Iliac crest autologous bone grafting (ICBG) has been the “gold standard” choice for autologous grafts because of its structural lattice that facilitates cell migration, proliferation, and tissue regeneration, using growth factors and osteoprogenitor cells [85]. However, ICBG has several disadvantages, including postoperative donor site pain, extended operating time, high intra-operative blood loss, the risk of infection, and limited availability of graft, particularly in elderly individuals [86]. Thus, BMPs have been considered as a replacement for ICBG. Among the several recombinant forms of BMP, the US Food and Drug Administration (FDA) has sanctioned two recombinant human (rh)BMPs: rhBMP-2 and rhBMP-7 [also known as osteogenic protein-1]. rhBMP-2 has been approved for use in the single-level anterior lumbar interbody fusion. At present, rhBMP-2 is marketed as an absorbable collagen sponge (ACS) that functions as a carrier for the protein. In contrast, rhBMP-7 has been approved as an alternative to autografts in compromised patients through the Humanitarian Device Exemption process. Two clinical trials compared rhBMP-2/ACS treatment against the standard ICBG for anterior lumbar interbody fusion procedures, reporting higher fusion rates for the rhBMP-2/ACS group [87,88]. Recently, a meta-analysis, conducted by the Yale University Open Data Access project, reported that rhBMP-2 enhanced the fusion rates in spinal surgery, compared to ICBG; however, it also highlighted concerns associated with the safety of rhBMP-2 [89].

### 5.3. Side Effects

The efficacy of BMPs resulted in their frequent “off-label” use in spinal fusion procedures [90]. However, the rapid increase in their use led to the emergence of a series of reports regarding the possible side effects of BMPs, including inflammation, ectopic/heterotopic bone formation, dysphagia in cervical spinal fusions, and vertebral bone resorption (osteolysis) [91,92]. Eventually, the 2008 FDA Public Health Notification published an alert regarding safety concerns for BMPs, which led to a gradual decline in their use. Therefore, despite having excellent osteoinductive capabilities, a series of potential side effects have restricted the widespread use of BMPs. The side effects of BMPs’ could be attributed to the administration of high-dose BMPs to induce sufficient fusion because of the degradation and rapid dilution (burst release) of these cytokines [93]. In addition, BMPs exhibit a dose-dependent efficacy [94]; however, their side effects are also dose-dependent [95], causing a dilemma in selecting an optimal dose. Therefore, enhancing the potency of BMPs and decreasing the use of high-dose BMPs would be clinically imperative.

### 5.4. Experimental Trials to Both Enhance the Anabolic Effect and Reduce the Side Effects of BMPs

The combined administration of PTH1-34 and BMP has been attempted to promote bone remodeling and lessen the amount of BMP dosage required and, thus, lowering the risk (and even preventing) the previously reported side effects of BMPs. Morimoto et al. [96] reported the positive effect of intermittent PTH1-34 administration on BMP induced bone formation in a rat model of spinal fusion. The fusion rate and bone volume density of newly formed bone in the group treated with BMP significantly increased with the concomitant administration of PTH1-34. Kaito et al. [97] also confirmed the modeling and remodeling effects of intermittent administration of PTH1-34 on BMP induced bone in a rat model of spinal fusion, and showed that PTH1-34 administration significantly decreased the tissue volume of the fusion mass at 12 weeks postoperatively, compared to 2 weeks postoperatively. According to an additional histomorphometric analysis of the cortical bone, periosteal bone resorption and endosteal bone formation were prominent.

In addition, several studies have reported that heterodimers, which are distinctive BMP family members are more potent than their constituent homodimers in inducing bone formation; in fact, BMP-2/6, -2/7 and -4/7 heterodimers have been shown to have a higher specific activity than their constituent homodimers [98,99,100]. Based on the sequence homology, BMPs are categorized into subfamilies. There are class I BMPs, comprising BMP2 and BMP4, and class II BMPs, comprising BMP5-8, and class I BMPs can form heterodimers with class II BMPs [101].

To date, although the underlying mechanism of the higher bone induction ability of BMP heterodimers remains partially understood, several clarifications have been suggested. First, heterodimers constitute a more stable receptor–ligand complex. Compared to BMP-2 or -6 homodimers [102], the BMP2/6 heterodimer exhibits a higher affinity to BMPRI and BMPRII, as well as a high SMAD1-dependent signaling activity. Second, heterodimers can better upregulate BMP receptor genes. Reportedly, BMP2/6 induces the expression of the BMPRII gene more effectively than BMP-2 or -6 homodimers [100]. Third, hetero- and homo-dimeric BMPs vary in their ability to control the synthesis of BMP inhibitors or are differentially affected by these inhibitors. For example, BMP2/7 heterodimers are not antagonized by Noggin, one of the soluble BMP antagonists, compared to BMP homodimers [103]. Perhaps the weaker Noggin antagonism on BMP heterodimers might contribute to the enhanced osteogenic potency of heterodimers, compared to that of homodimers. Although these results suggest that BMP heterodimers could be an alternative to BMP homodimers, whether lower doses of BMP heterodimers result in bone formation to the same extent as BMP homodimers, while reducing the secondary inflammatory response, remains unclear [95].

### 5.5. Carrier Materials for Delivering BMPs

To date, various materials have also been assessed to enhance the delivery of BMPs. Carrier materials are classified into four main types: natural polymers, synthetic polymers, inorganic materials (mainly ceramics), and their composites [104]. Each class provides some advantages and disadvantages. Currently, the trend is to use composite carriers that provide the benefits of each class of materials. For example, semisynthetic polymers, which exhibit controlled release properties, were introduced due to their biocompatibility in combining with natural polymers, including polycaprolactone within collagen [105], PEGylated fibrinogen [106]. In another example, composites, that combined collagen to biphasic calcium phosphate, were superior to biphasic calcium phosphate alone for bone regeneration, while decreasing the incidence of burst release [107]. Therefore, developing an ideal carrier material for delivering BMPs that can localize the protein, prolong its retention time at the site of action and provide mechanical strength and a scaffold for bone ingrowth is essential.

## 6. Anti-Sclerostin Antibody

### 6.1. Mechanism of Action

Sclerostin, which is the product of the SOST gene, is a negative regulator of bone formation [108,109,110]. Sclerostin is considered to be mainly produced from osteocytes, although its messenger ribonucleic acid has also been detected in chondrocytes, the kidney, lung, liver, vascular tissue, and the heart [111,112,113]. Sclerostin works as an antagonist of BMP and Wnt signaling, with the main function of sclerostin on bone metabolism being the inhibition of the Wnt/β-catenin pathway in osteoblasts via binding to the low-density lipoprotein receptor-related protein 5/6 receptor on the membrane of osteoblasts [114,115,116]. Thus, the inhibition of sclerostin can induce the activation of osteoblasts and promote bone formation (Figure 1 and Figure 2). For therapeutic use in humans, the anti-sclerostin antibody (romosozumab) has been developed for the treatment of osteoporosis, which decreases endogenous levels of sclerostin, allowing for osteogenesis through an improvement in osteoblast survival [117,118,119]. Several authors have reported on the anabolic effect of anti-sclerostin antibody in enhancing the bone formation and fracture healing in animal models [120,121,122,123,124]. In addition to the anabolic effect of anti-sclerostin antibodies, Suen et al. [122] suggested that anti-sclerostin antibodies could also induce an early increase in neovascularization around the fracture site, which would also contribute to enhanced fracture healing. 

### 6.2. Experimental Studies in Animal Spinal Fusion Models and Clinical Trials for Human Spinal Fusion

There have been no studies on the use of anti-sclerostin antibodies for animal or human models of spinal fusion to date. However, taking into consideration the anabolic effect of anti-sclerostin on bone metabolisms and its efficacy in the treatment of osteoporosis, there is a promise that anti-sclerostin could enhance spinal fusion. 

## 7. Prostaglandins Agonist

### 7.1. Mechanism of Action

Prostaglandins (PGs) have not only a stimulatory but also an inhibitory effect for bone metabolism depending on the physiological or pathological conditions. In particular, PGE2 produced by osteoblasts under cyclooxygenase (COX)-2 stimulation plays an important role in bone metabolism [125]. There are four subtypes of receptors for PGE2 (EP1, EP2, EP3, EP4), and studies have shown signaling, via EP2 and EP4, to play an important role in bone metabolism [126]. With regard to the anabolic aspect of bone metabolism, PGE2 induced the expression of the core-binding factor alpha1 (Runx2/Cbfa1) and enhanced the mineralized nodule formation. These phenomena could not occur in the culture of cells from EP4-deficient mice (Figure 1 and Figure 2) [127]. In several animal and human studies of spinal fusion, non-specific non-steroidal anti-inflammatory drugs have been shown to exert a strong negative effect on the rate of spinal fusion, though there is no consensus about the effects of COX-2 inhibitors on spinal arthrodesis [126,128]. With regard to therapeutic trials, many studies have focused on EP4 receptor activation, demonstrating the effectiveness of EP4 agonist for fracture healing and osteoporosis in animal models [129,130,131,132].

### 7.2. Experimental Studies in Animal Models of Spinal Fusion and Issues for Clinical Use in Human Spinal Fusion

Namikawa et al. [131] revealed that the local administration of an EP4 receptor agonist promoted the osteoinductivity of BMP-2 in a rabbit posterolateral lumbar spinal fusion model. Recently, Kanayama et al. [133] showed that an IP (PGI2 receptor) agonist also promoted osteoblast differentiation and ectopic and orthotopic bone formation in vivo in a rat model of spinal fusion. PG receptor agonists may induce several specific side effects (such as local and/or systemic inflammation, hypotension, tachycardia, and diarrhea) and, thus, further research is required to elucidate these side effects for human clinical use. However, PG agonists may provide a therapeutic potential to enhance bone fusion in spinal arthrodesis.

## 8. Cell Therapies

### 8.1. Mechanism of Action and Cell Sources

Cell-based therapies, which aim to enhance osteogenesis, osteoconduction, and/or osteoinduction of bone graft, have been tried for spinal arthrodesis. Mesenchymal stem cells (MSCs) are key cells for these therapies and have been widely used to promote bone formation and regeneration in many animal and human trials [134,135]. Bone marrow-MSCs (BM-MSCs), isolated by bone marrow aspiration from the iliac crest or vertebral body, are considered to be suitable for spinal arthrodesis due to their intra-operative accessibility [136,137]. MSCs are multipotent stem cells that have the capability for self-renewal, plasticity, and multilineage potential, including osteogenic, chondrogenic, adipogenic, and myogenic lineages. Their differentiation relies on both intrinsic and extrinsic factors in their environments, where BMP signaling has an important role in the differentiation of MSCs to osteoblasts by activation of Runx2, via SMAD1/5/8 [14,15,16,138]. Adipose-derived stem cells (ASCs) have also been attracting attention as a source of MSCs. ASCs are attractive because they are easily accessible and adipose tissue has a high cellular content, but also, ASCs have a higher capacity for self-renewal and plasticity than BM-MSCs [134].

### 8.2. Experimental Studies in Animal Spinal Fusion Models, Clinical Trials for Human Spinal Fusion and Issues for Clinical Use

In an animal posterolateral spinal fusion model with rabbits, Nakajima et al. [139] reported that successful fusion observed in four of 5 rabbits with cultured osteogenic BM-MSCs in Type-1 collagen gel, compared to none of 6 rabbits with hydroxyapatite in Type-1 collagen gel. Yang et al. [140] also evaluated the effectiveness of osteogenic BM-MSCs in the rabbit anterior lumbar interbody fusion model. Four of 10 rabbits with porous collagen sponge with cultured osteogenic BM-MSCs and 7 of 10 rabbits with iliac crest bone graft achieved bony fusion, compared to none of 10 rabbits without bone graft or collagen sponge. Against these experiments from animal studies, several cellular bone matrices, which are allogenic bone grafts containing living MSCs, have been commercially available for human spinal arthrodesis. Though no randomized controlled trial has been conducted to evaluate the efficacy of cellular bone matrices in spinal arthrodesis and there is no evidence that MSCs can survive, differentiate, and regenerate after being transplanted into the human spinal fusion site, they can be a promising alternation for bone augmentation [141]. However, potential long-term drawbacks, such as mal-differentiation and tumorigenicity, are of concern and still need to be solved before MSCs can be used for clinical purposes in humans [142]. Instead of MSCs, bone marrow aspiration (BMA) combined with synthetic or allograft materials has been used to enhance bony fusion after spinal arthrodesis in clinical practice because they contain different cell populations including osteoprogenitors and hematoprogenitors [135,143]. One concern is that BMAs harvested from the iliac crest contains only one to five MSCs per 500,000 nucleated cells [141,144]. This could make the therapeutic effect of BMA uncertain. Moreover, there is no clear evidence that BMA combined with synthetic or allograft materials can be a substitute or supplementary graft to autologous bone [135,145]. Yousef et al. [145] reported that a collagen scaffold with BMA, using selective cell retention technology for the intraoperative concentration of MSCs, could lead to successful fusion and improved clinical outcomes after human posterolateral fusion [145]. Recently, it has been demonstrated that induced pluripotent stem (iPS) cells can be differentiated into osteoblasts and this technology may alter the strategy for bone regenerative cell therapies [146]. Several high-quality comparative studies aiming to reveal the efficacy of cell therapies are ongoing, and their evidence will be clarified in the future [134,135,141].

## 9. Gene Therapies

The main limitation of using osteogenic agents, such as BMPs, for inducing bone formation after spinal arthrodesis is the need for safe and effective drug delivery systems that will provide a sustained and biologically appropriate concentration of the osteogenic factor at the target sites [134]. Gene therapy approaches aim to deliver osteoinductive genes locally to induce bone formation and improve spinal fusion, with several approaches having been tried [134]. The vectors used for gene therapy approaches consist of an adenoviral vector, lentiviral vector, naked deoxyribonucleic acid, liposomes, and plasmids. Not only BMPs, but also Nell-like molecule, LIM mineralization protein, and SMAD1 have been tested as transduction genes to enhance the spinal fusion in animal models [98,134,147,148,149]. The main problems with genetic engineering are cell toxicity, immunization, insertional mutagenesis, and low cell selectivity. Thus, though further studies are still needed for the clinical application of gene therapies in spinal fusion, these therapies do hold the promise for eliminating the use of autograft and its associated morbidities.

## 10. Overview and Future Direction

In addition to anti-bone resorptive agents which have been widely used so far, the recent progress in bone pharmacophysiology provides us with newly developed bone anabolic agents. These agents certainly have an advantage over anti-bone-resorptive agents to enhance bone fusion after spinal fusion surgery. Furthermore, cell and gene engineering techniques have a great potential to make innovative changes in drug delivery systems or environment for bone formation. These attempts for biological enhancement of bone formation can offer a reliable fusion after spinal fusion or shorten the period for achieving bone fusion. The development of artificial bone grafts, using osteoinductivity and osteoconductivity, may reduce the necessity of harvesting autologous bone graft and the incidence of related complications. The limitation of previous experimental studies was that many studies about the biological enhancement for bone formation after spinal fusion were performed using small animals such as a rat, mouse, and rabbit. Pharmacokinetic and local environments around the fusion site can be different between such small animals and a human. The evaluation with large animals or establishment of experimental models closer to the human environment with small animals is desirable in the future. In particular, the establishment of the interbody fusion model in small animals is difficult because the vertebral endplates can be easily sacrificed and thus local environment in human interbody fusion cannot be reproduced. Recently, we have established a rat interbody fusion model without violating endplates and demonstrated the reproducibility of BMP-2 related dose-dependent complications such as soft tissue swelling and osteolysis (unpublished data). Furthermore, several issues remain to be solved particularly with regard to the safety and cost-effectiveness of such novel approaches for human clinical use. However, we believe that the development of the biological enhancement of spinal fusion would be of benefit to patients’ health-related QOL outcomes, as well as being important for social health economics.

## Figures and Tables

**Table 1:** The summary of approaches for biological enhancement of spinal fusion identified in this review.

	Mechanism of Action	Effect on Bone Metabolism	Clinical Trials for Human Spinal Fusion	Effect on Fusion in Animal Models	Effect on Fusion in Human
Bisphosphonates	Involved in osteoclasts and induction of apoptosis of osteoclasts	Inhibition of bone resorption	Yes	Yes	Controversial
Anti-RANKL monoclonal antibody	Prevention of the interaction between RANKL and RANK receptor on osteoclasts and osteoclast precursors by binding RANKL	Inhibition of bone resorption	No	N/A	N/A
PTH1-34	Stimulation of osteoblast differentiation by intermittent PTH (PTH1-34)	Activation of bone formation (intermittent PTH1-34) Activation of bone resorption (continuous PTH1-34)	Yes	Yes	Yes
BMPs	Activation of Runx2 expression and induction of osteoblast differentiation	Activation of bone formation	Yes	Yes	Yes
Anti-sclerostin antibody	Inhibition of sclerostin which interferes BMP and Wnt signaling	Activation of bone formation	No	N/A	N/A
Prostaglandins agonist	Activation of Runx2 expression	Activation of bone formation	No	Yes (combined use with BMP)	N/A
Stem cell	Induction of mesenchymal stem cells (bone marrow stem cells, adipose-derived stem cells, and bone marrow aspiration)	Supplementation of cell source for osteoblast	Yes	Yes	Yes
Gene therapy	Delivery of osteoinductive genes locally around the sites of fusion	Activation of bone formation	No	N/A	N/A

RANKL indicates Receptor activator of nuclear factor κB ligand; N/A, not available; PTH, parathyroid hormone; BMP, bone morphogenetic protein; Runx2, Runt-related transcription factor-2.

## Abbreviations

ACS	absorbable collagen sponge
ASC	adipose derived stem cell
BMA	bone marrow aspiration
BMD	bone mineral density
BM-MSC	bone marrow-mesenchymal stem cell
BMP	bone morphogenetic protein
BRONJ	bisphosphonate-related osteonecrosis of the jaw
Cbfa1	core-binding factor subunit alpha-1
COX	Cyclooxygenase
FDA	the US Food and Drug Administration
ICBG	iliac crest autologous bone grafting
OPG	Osteoprotegerin
PG	Prostaglandin
PKCδ	protein kinase Cδ
PTH	parathyroid hormone
QOL	quality of life
RANKL	receptor activator of nuclear factor κB ligand
rhBMP	recombinant human BMP
Runx2	Runt-related transcription factor 2
